# N-Terminal Pro Brain Natriuretic Peptide, sST2, and Galectin-3 Levels in Breast Cancer Survivors

**DOI:** 10.3390/jcm10153313

**Published:** 2021-07-27

**Authors:** Shruti Rajesh Patel, Joerg Herrmann, Robert A. Vierkant, Janet E. Olson, Fergus J. Couch, Antonious Hazim, Jeff A. Sloan, Charles L. Loprinzi, Kathryn J. Ruddy

**Affiliations:** 1 Department of Internal Medicine, Mayo Clinic, Rochester, MN 55901, USA; hazim.antonious@mayo.edu; 2 Department of Cardiovascular Disease, Mayo Clinic, Rochester, MN 55901, USA; Herrmann.Joerg@mayo.edu; 3 Health Sciences Research, Mayo Clinic, Rochester, MN 55901, USA; vierkant.robert@mayo.edu (R.A.V.); olsonj@mayo.edu (J.E.O.); jsloan@mayo.edu (J.A.S.); 4 Department of Laboratory Medicine and Pathology, Mayo Clinic, Rochester, MN 55901, USA; Couch.Fergus@mayo.edu; 5 Department of Oncology, Mayo Clinic, Rochester, MN 55901, USA; cloprinzi@mayo.edu (C.L.L.); Ruddy.Kathryn@mayo.edu (K.J.R.)

**Keywords:** breast cancer, anthracycline toxicity, cardio-oncology

## Abstract

NT-proBNP, soluble ST2 (sST2), and galectin-3 are biomarkers of cardiac dysfunction that have been proposed as identifiers of patients experiencing asymptomatic cardiac dysfunction after anthracycline-based chemotherapy. This study aimed to compare the proportion of breast cancer (BC) survivors with elevated serum levels of these three putative biomarkers by prior receipt of anthracycline (yes vs. no). Five-hundred-eighty survivors of BC who had received anthracycline-based chemotherapy were matched by age and time between diagnosis and serum storage to 580 who had not. Cardiac biomarker levels were analyzed using immunoassays. Analyses were carried out using linear and logistic regression models. Anthracycline recipients had higher values of NT-proBNP than non-recipients (mean 116.0 ng/L vs. 97.0 ng/L, respectively; *p* < 0.001). Values for ST2 and galectin-3 did not significantly differ by receipt of anthracycline. After further adjustment for age at breast cancer diagnosis, ethnicity, and receipt of trastuzumab, associations between receipt of anthracycline and higher NT-proBNP persisted (*p* < 0.001), showing that NT-proBNP may be a biomarker of cardiovascular toxicity after receipt of anthracycline-based chemotherapy. Further research to assess the clinical utility of NT-proBNP testing after receipt of anthracycline is recommended. sST2 and galectin-3 do not appear to differentiate between anthracycline recipients and non-recipients amongst breast cancer survivors.

## 1. Introduction

Cardiovascular diseases are among the leading contributors to mortality in cancer survivors, accounting for 30–50% of all deaths in non-metastatic breast cancer (BC) survivors over the age of 65 [1]. This appears to be, in part, due to use of anthracycline-based chemotherapy in the neoadjuvant or adjuvant setting. Reduced left ventricular ejection fraction (EF) is the hallmark of anthracycline cardiotoxicity, and some studies have identified post-anthracycline right ventricular dysfunction as well [2]. A study using data from the Surveillance, Epidemiology, and End Results (SEER) registry regarding non-metastatic breast cancer patients over the age of 65 demonstrated a 19% incidence of heart failure, defined as a decrease in EF causing symptoms within three years of treatment with an anthracycline [3]. A cross-sectional study of a slightly younger breast cancer population (median age 63) assessed ejection fraction more than five years after diagnosis (81% of whom had received anthracycline) and found a 15% rate of reduced EF [4]. Especially if undertreated, the prognosis of symptomatic anthracycline-induced cardiomyopathy is poor [5,6]. Screening initiatives to identify and treat anthracycline-induced cardiotoxicity before it becomes symptomatic might help improve long-term outcomes not only after breast cancer treatment, but also for survivors of other cancers (e.g., lymphoma, leukemia, and sarcoma) for which anthracyclines are commonly administered.

Anthracycline-induced cardiotoxicity is thought to be mediated by topoisomerase II β, interrupting mitochondrial biogenesis, generating reactive oxygen species and double-strand DNA breaks, and subsequently activating apoptosis of cardiac myocytes [7]. This process is thought to be largely irreversible, and once symptoms of heart failure develop, 5-year survival has historically been less than 50% [3,6]. Although anthracycline use has been falling in recent years due to the availability of alternative, less cardiotoxic drugs, a 2012 Medicare study showed that 32% of patients with breast cancer still received anthracycline-based regimens at that time [1].

Cardiac biomarkers such as natriuretic peptides, including N-terminal prohormone brain-type natriuretic peptide (NT-proBNP), can be elevated due to cardiac stress prior to symptom onset; thus, these may be useful for risk stratification in order to help target cardiovascular interventions to those most likely to benefit. The 2016 European Society of Cardiology Guidelines recommend natriuretic peptide testing for all patients with suspected heart failure (HF) and one additional factor of heightened probability, including prior exposure to cardiotoxic medications such as anthracyclines [8]. A recent retrospective evaluation of adult survivors of childhood cancers with exposure to cardiotoxic therapeutics demonstrated abnormal NT-proBNP levels and an increased risk of future cardiomyopathy [9]. A diagnostic cutoff point at the upper limit of normal (125 ng/L for NT-proBNP) has been suggested for triaging which patients may benefit from echocardiography. Importantly, BNP levels predict mortality risk even in patients without HF (but with other cardiovascular diseases) [10]. Studies have shown that anthracycline-treated breast cancer patients with persistently elevated NT-proBNP levels had significantly worse LV impairment than anthracycline-treated patients with normal or transiently elevated NT-proBNP levels. [11] While the mechanisms of this are not completely understood, it is theorized that both cardiovascular and hemodynamic stress from other medical conditions may contribute to higher natriuretic peptide levels [10]. This phenomenon is seen in breast cancer patients exposed to other cardiotoxic agents such as trastuzumab, which has been shown to cause a higher risk of developing cardiotoxicity in patients whose NT-proBNP levels were above the upper limit of the normal range. [12] While the American Heart Association (AHA) guidelines provide a class IIa recommendation for the measurement of natriuretic peptides in patients at risk of HF, the current American Society of Clinical Oncology (ASCO) guidelines (published in 2017) and National Comprehensive Cancer Network (NCCN) Survivorship guidelines (updated annually) do not recommend this routinely, instead suggesting that cancer survivors who have one or more clinical cardiac risk factors (i.e., smoking, hypertension, diabetes, dyslipidemia, obesity, age ≥ 60) undergo an echocardiogram 6–12 months after anthracycline completion (without biomarker assessment beforehand) [13].

Two other emerging biomarkers for HF: suppressor of tumorgenicity 2 (sST2) and galectin-3, have been shown to independently correlate with the risk of hospitalization and death in patients with known HF [14]. sST2 is a soluble protein released with myocardial strain and plays an important role in myocardial remodeling [15]. Importantly, serial measurements of sST2 levels also independently predict reverse left ventricular remodeling [16]. Galectin-3 is a lectin that is upregulated in HF and appears to play an important role in left ventricular remodeling. Galectin-3 has been found to be useful in predicting mortality in patients with HF in conjunction with NT-proBNP [17], but NT-proBNP and sST2 are likely superior to galectin-3 for prognostication purposes in patients with chronic HF [18]. Interestingly, there is some evidence that galectin-3 expression may also augment cancer growth, invasiveness, and metastatic potential, and possibly could help the tumor evade immune surveillance [19,20]. Testing for both sST2 and galectin-3 are considered class II recommendations for risk prediction in heart failure from the American College of Cardiology, American Heart Association, and Heart Failure Society of America Heart Failure guidelines [14,21], but are not included in ASCO or NCCN guidelines.

This study aimed to address a gap in knowledge by comparing levels of NT-proBNP, sST2, and galectin between breast cancer survivors previously treated with anthracycline-based chemotherapy and survivors with no prior anthracycline exposure.

## 2. Methods

### 2.1. Study Design

This retrospective cohort study compared cardiac biomarkers by prior receipt of anthracycline using stored serum from women who had a previous breast cancer diagnosis (participants in a longitudinal cohort study). In order to minimize confounding, each patient who had received anthracycline was matched to a non-recipient based on age and time from cancer diagnosis to blood draw. Specifically, there was a 1:1 match by age (within 5 years) and by time between breast cancer diagnosis and sample collection (within 6.5 months). This study was approved by the Mayo Clinic Institutional Review Board (IRB #1815-04).

### 2.2. Study Population

The Mayo Clinic Breast Disease Registry (MCBDR) aims to enroll all patients seen at least once at the Mayo Clinic in Rochester, Minnesota within a year of a new breast cancer diagnosis; after informed consent, participants are asked to provide serial blood and survey, as well as access to stored tumor tissue. Blood samples were obtained by venipuncture. After adequate centrifugation, serum samples were stored at −80 °C. Five-hundred-eighty-one female patients who had received anthracycline-containing chemotherapy and 581 who had not were identified from the MCBDR database. One matched pair was excluded because one of the subjects was a male patient; this resulted in 580 matched pairs for analysis, or 1160 subjects overall. Receipt of anthracycline was confirmed by MD review.

### 2.3. Biomarker Assessment

NT-proBNP levels were assessed using an immuno-electrochemiluminescence assay (Roche Diagnostics, Hitachi High Tech Corp, Indianapolis, IN, USA). Soluble ST2 was determined using a high-sensitivity sandwich monoclonal immunoassay (Presage ST2 assay, Critical Diagnostics, San Diego, California, CA, USA), and galectin-3 levels were assessed using an enzyme-linked fluorescent assay (BioMerieux ref. 411191). Values greater than the upper limit of detection were set to the highest value detectable; galectin-3 values greater than 100 ng/L were set to 100, and sST2 values greater than 200 ng/L were set to 200. NT-proBNP values less than 10 ng/L were set to 10, and values greater than 2000 ng/L were set to 2000.

### 2.4. Statistical Methods

Data were summarized using frequencies and percentages for categorical variables, and means and standard deviations for continuous variables. Comparisons of demographic, clinical, and biomarker attributes with receipt of anthracycline were carried out using conditional logistic regression for categorical variables and linear regression models for continuous variables. For each, the attribute of interest was modeled as the outcome (y-) variable and anthracycline receipt was modeled as the exposure (x-) variable. All analyses accounted for the matched nature of the data by including a matched set ID in the model. For the logistic models, this was carried out using the standard conditional likelihood approach. For the linear regression models, set ID was included as a blocking factor. We first examined univariate associations by fitting separate regression models for each attribute, in turn. These analyses are mathematically equivalent to McNemar tests for categorical variables (when using the score test from the logistic model) and paired *t*-tests for continuous variables. Following the univariate analyses, we fitted multivariate models to assess associations between attributes and anthracycline receipt after accounting for the effects of the other attributes. Finally, we examined associations of biomarker values with age at breast cancer diagnosis and time from diagnosis to serum collection in the pooled set of anthracycline recipients and non-recipients, using Pearson correlation coefficients. Due to the inherent right skewness in the biomarker data, regression analyses were carried out using rank-transformed scores, with values for the 1160 subjects ranging from 1 (for the lowest original/raw score) to 1160 (for the highest original/raw score). Sensitivity analyses were run using original values, log-transformed values, and inverse normal (van der Waerden) rank scores. All statistical tests were two-sided, and all analyses were carried out using the SAS System (SAS Institute, Inc., Cary, NC, USA).

## 3. Results

The mean age at breast cancer diagnosis for the 1160 participants (half of whom were prior anthracycline recipients) was 52.2 years (SD 9.6), 94% identified their race as White, and 92% identified their ethnicity as non-Hispanic. The stored serum used to assess cardiac biomarkers was collected on average 2.7 years after diagnosis of breast cancer (SD 1.7). Comparisons of demographic and clinical variables by receipt of anthracycline are shown in Table 1. Although the study design matched on age at diagnosis, anthracycline recipients were on average 0.3 years younger at the time of the serum collection than paired non-recipients of anthracycline (*p* < 0.001). Anthracycline recipients were much more likely to have received trastuzumab than non-recipients (29.3 vs. 7.9%, respectively; *p* < 0.001). Anthracycline recipients were also more likely to identify as non-Hispanic than non-recipients (*p* < 0.001). There were no differences in time from breast cancer to serum collection between anthracycline recipients and non-recipients.

Comparisons of cardiac serum biomarkers by anthracycline receipt are shown in Table 2. On average, recipients had higher values of NT-proBNP (mean 116.0 ng/L) than non-recipients (97.0, *p* < 0.001). Values for sST2 and galectin-3 did not significantly differ by receipt of anthracycline (*p* = 0.78 and 0.86, respectively, Table 2). After further adjustment for age at breast cancer diagnosis, ethnicity, and receipt of trastuzumab, associations between the receipt of anthracycline and NT-proBNP values persisted (*p* < 0.001), while associations with sST2 and galectin-3 remained non-significant (Table 2). Sensitivity analyses replacing biomarker ranked values with original values, log-transformed values, or inverse normal van der Waerden ranks did not substantively change the results (data not shown). Anthracycline recipients were also more likely to have NT-proBNP > 125 ng/L (N = 163, 28%) than non-recipients (N = 119, 21%, *p* = 0.002).

**Figure 1:**
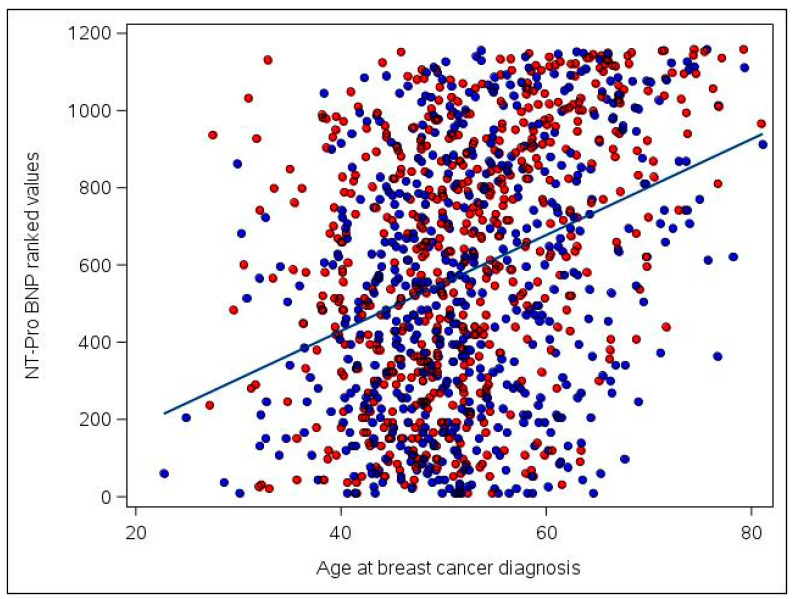
Association of ranked NT-proBNP values with age at breast cancer diagnosis among the 1160 participants included in the study. Ranked NT-proBNP values range from 1 (for the participant with the lowest original/raw NT-proBNP value) to 1160 (for the participant with the highest original/raw value). Anthracycline users are plotted in red and non-anthracycline users are plotted in blue. A least squares regression line is superimposed on the figure for ease of interpretation. Correlation = 0.36 (*p* < 0.001).

Further examination of the NT-proBNP biomarker in the pooled set of anthracycline recipients and non-recipients found that values tended to modestly increase as time from diagnosis to blood draw increased (correlation r of ranked NT-proBNP values with time = 0.06, *p* = 0.05). This association attenuated to null after accounting for age at diagnosis (partial correlation coefficient r = 0.04, *p* = 0.13). Correlations between higher NT-proBNP and older age at diagnosis were stronger (r = 0.36, *p* < 0.001, Figure 1).

## 4. Discussion

As symptomatic cardiotoxicity from cancer-related therapies can result in treatment interruptions, hospitalizations, morbidity, and mortality, and because there are medications that can slow or prevent the development of symptomatic HF, biomarkers that identify asymptomatic cardiac dysfunction may be of value in cancer survivors.

In this relatively large, single-center, retrospective analysis evaluating cardiac biomarkers in patients with breast cancer, the following was observed: (1) anthracycline recipients had higher values of NT-proBNP than non-recipients; (2) increasing time between breast cancer diagnosis and serum collection was associated with higher NT-proBNP values; and (3) sST2 and galectin-3 did not differ between anthracycline recipients and non-recipients.

### 4.1. Natriuretic Peptide

The role of natriuretic peptides for the detection of myocardial injury after antineoplastic therapy has been studied in a variety of patient populations and at various timepoints [22,23,24,25,26,27]. A 2019 study demonstrated that NT-proBNP levels are accurate predictors of cardiotoxicity and can identify different risk levels in patients that received doxorubicin-containing chemotherapy [28]. In addition, studies have identified that NT-proBNP is temporally associated with changes in LVEF in breast cancer survivors [29,30]. Despite this, the use of NT-proBNP as a biomarker after anthracycline receipt remains controversial, and is not included in NCCN or ASCO guidelines.

The current results demonstrate that patients with exposure to anthracyclines have higher NT-proBNP levels than non-recipients. Ranked analysis confirmed that values for NT-proBNP in anthracycline recipients were higher than non-recipients, and that this association persisted after adjustment for age at breast cancer diagnosis, ethnicity, and receipt of trastuzumab. Furthermore, the detection of abnormal NT-proBNP levels (>125 ng/L) in almost a third of anthracycline recipients in the current study correlates well with data from children who had previously received anthracyclines [31]. As expected, increasing age was positively associated with NT-proBNP levels, and we found a modest association between greater time from breast cancer diagnosis to serum collection and higher NT-proBNP levels. The current study findings suggest that NT-proBNP may be a useful biomarker of myocardial injury in asymptomatic cancer survivors who have previously received an anthracycline, though additional research is needed to investigate clinical correlates of NT-proBNP levels in this setting.

### 4.2. Soluble ST2

The ST2 gene is upregulated in the setting of myocardial stretch, increasing levels of soluble ST2 rapidly. However, the current study demonstrated that values of sST2 were not significantly different between the patients who received anthracyclines and those who did not. This supports that sST2 is not useful as an early biomarker of cardiac dysfunction from anthracyclines. These results are concordant with those of a study comparing sST2 in survivors of adult-onset lymphoma and childhood cancer to sST2 in healthy controls [32,33].

### 4.3. Galectin-3

Galectin-3 is a known marker of cardiac fibrosis; higher levels of this protein have been previously shown to be associated with an increased risk of heart failure and all-cause mortality in community populations, as well as with risk of morbidity and mortality amongst patients with heart failure. The absence of a difference in galectin-3 levels between anthracycline recipients and non-recipients suggests that galectin-3 is likely not a useful biomarker of cardiotoxicity after anthracycline receipt for breast cancer.

This study has several limitations including its retrospective design and single-center study population. In addition, we did not assess levels of cardiac troponin, which has previously been shown to be a promising biomarker of myocardial injury from oncologic therapy [34,35,36]. In addition, our samples were frozen for many months prior to biomarker assessment, which could have impacted the results. Published data have demonstrated the long-term stability of endogenous NT-proBNP, sST2, and galectin-3 for 4 months, 18 months, and 3 months, respectively, but little is known about stability over longer periods of time [37,38,39]. It is also important to note that there is overlap between the pathophysiology of CVD and cancer, including inflammation and cellular proliferation [40], which may affect the utility of these biomarkers for cardiovascular disease detection amongst cancer survivors in clinical practice. In addition, the fact that galectin-3 levels can be elevated due to liver cirrhosis and pulmonary fibrosis and that sST2 can be elevated due to diabetes and hypertension may have impacted the results as well [15,41]. Future studies investigating the correlation of these biomarkers with cardiac risk factors (i.e., smoking history, hypertension, diabetes mellitus, etc.) and echocardiographic parameters, including global longitudinal strain, would enhance our understanding of the clinical relevance of our findings. Still, we conclude that NT-proBNP may be a biomarker of cardiovascular toxicity after receipt of anthracycline-based chemotherapy, and future research should further assess the clinical utility of NT-proBNP testing after receipt of anthracycline (including at what timepoints this is most informative). sST2 and galectin-3 do not appear to differentiate between anthracycline recipients and nonrecipients amongst breast cancer survivors.

## Figures and Tables

**Table 1:** Comparison of demographic and clinical variables with receipt of anthracycline.

-	Did Not Receive Anthracycline (*n* = 580)	Received Anthracycline (*n* = 580)	Comparison ^1^	*p*-Value ^2^
Age at breast cancer diagnosis in years, mean (SD)	52.4 (9.5)	52.0 (9.7)	−0.3 (−0.4, −0.2)	<0.001
Time from breast cancer diagnosis to blood draw in years, mean (SD)	2.7 (1.7)	2.7 (1.7)	0.01 (−0.01, 0.01)	0.20
-	-	-	-	-
Race, N (percent)	-	-	-	0.71
White	546 (94.1)	549 (94.7)	1.00 (ref)	-
Other/unknown	34 (5.9)	31 (5.3)	1.10 (0.67, 1.80)	-
-	-	-	-	-
Ethnicity, N (percent)	-	-	-	<0.001
Not Hispanic or Latino	514 (88.6)	549 (94.7)	1.00 (ref)	-
Other/unknown	66 (11.4)	31 (5.3)	2.35 (1.48, 3.72)	-
-	-	-	-	-
Receipt of trastuzumab, N (percent)	-	-	-	<0.001
No	534 (92.1)	410 (70.7)	1.00 (ref)	-
Yes	46 (7.9)	170 (29.3)	4.44 (3.10, 6.38)	-
-	-	-	-	-

N: number of subjects; SD: standard deviation; ^1^: values comparing anthracycline recipients to non-recipients. Mean difference in values with corresponding 95% confidence interval for continuous variables, and conditional logistic regression odds ratios with 95% confidence interval for categorical variables; ^2^: linear regression analysis modeling matched set ID as a categorical blocking factor for continuous variables, conditional logistic regression analysis for categorical variables.

**Table 2:** Comparison of serum-derived cardiac biomarkers with receipt of anthracycline.

-	Did Not Receive Anthracycline, Mean (SD)	Received Anthracycline, Mean (SD)	Mean Difference (95% CI) ^1^	*p*-Value ^2^	*p*-Value ^3^
NT-proBNP (ng/L)	97.0 (137.6)	116.0 (153.3)	19.0 (2.9, 35.1)	<0.001	<0.001
sST2 (ng/L)	25.1 (14.7)	25.0 (14.5)	−0.1 (−1.7, 1.63)	0.78	0.93
Galectin-3 (ng/L)	16.1 (7.8)	15.8 (7.5)	−0.3 (−1.2, 0.5)	0.86	0.97
-	-	-	-	-	-

SD: standard deviation; ng/L: nanograms per liter; ^1^: mean difference in values between anthracycline recipients and non-anthracycline recipients and corresponding 95% confidence interval; ^2^: linear regression analysis modeling matched set ID as a categorical blocking factor; ^3^: multivariate linear regression analysis simultaneously modeling NT-proBNP, sST2, galectin-3, age at breast cancer diagnosis, ethnicity and trastuzumab, and including matched set ID as a categorical blocking factor.

## Data Availability

The data presented in this study are available on request from the corresponding author. The data are not publicly available due to privacy protection of the participants.
